# An effective solution to simultaneously analyze size, mass and number concentration of polydisperse nanoplastics in a biological matrix: asymmetrical flow field fractionation coupled with a diode array detector and multiangle light scattering†

**DOI:** 10.1039/d1ra00450f

**Published:** 2021-04-06

**Authors:** Xing-ling Luo, Ying-ting Wu, Ling-yan Zhang, Ke-xin Li, Tian-jiang Jia, Yi Chen, Li-hong Zhou, Pei-li Huang

**Affiliations:** School of Public Health, Capital Medical University Beijing 100069 China; Core Facility Center, Capital Medical University Beijing 100069 China; School of Basic Medical Sciences, Capital Medical University Beijing 100069 China

## Abstract

To accurately understand the biological pollution level and toxicity of polydisperse nanoplastics, an effective solution is presented to separate polydisperse nanoplastics and detect their size, mass and number concentration in a biological matrix by asymmetrical flow field fractionation coupled with a diode array detector and a multiangle light scattering detector.

## Abstract 2

AF4-DAD-MALS is proposed to separate polydisperse nanoplastics and detect their size, mass and number concentration in a biological matrix.

## Graphical abstract

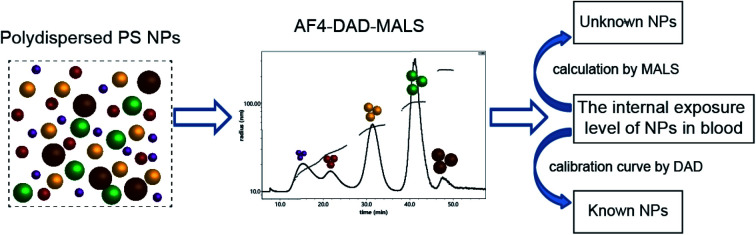

Nanoplastics (NPl, <1000 nm), produced from industrial nanomaterials or degraded from fragmentation of ubiquitous plastic products, are emerging nanopollutants.^1–3^ With the development of nanotechnology and the increase in the production and use of plastics, the possibility of direct or indirect human exposure to NPl has further increased.^4–8^ Numerous studies have reported adverse health effects from exposure to NPl, and indicated that the size of NPl is of crucial importance for their biological effects.^9–13^ In particular, small NPl can cross biological membranes including the blood–brain barrier, and show higher accumulation and adsorb higher concentrations of contaminants in comparison with lager particles.^14–16^ Recently, a study has pointed out that the original toxicity of small size NPl could not be reduced by aggregating them into large size particles.^17^ In addition, a dose-dependent increase of toxicity has been observed, indicating that the mass concentration of particles is related to the toxicity.^18^ Simultaneously, the quantity of particles, as essential information for studying nanomaterials according to the European Union, is relevant to the exposure assessment and pollution level.^19–21^ Therefore, it is vital to monitor differently sized NPl including their size, mass and number concentration in the biological matrix for accurately studying their toxicity and pollution level.

Current techniques for measuring NPl involve electron microscopy, dynamic light scattering (DLS) or nanoparticle tracking analysis (NTA) to detect its size, and pyrolysis-gas chromatography (Pyr-GC-MS), NTA, fluorescence spectrophotometer, Raman to determine its mass or number concentration.^6,22,23^ However, microscopy technologies extracting quantitative information of NPl through particle-by-particle characterizations and calculations, is inaccurate and time-consuming.^24^ DLS could not accurately measure the size of polydisperse particle due to its poor resolution.^25^ NTA requires sophisticated instruments, highly trained personnel, has limited size resolution which would be biased to larger particles for polydisperse sample.^20,26^ The Pyr-GC-MS or fluorescence spectrophotometer does not distinguish the mass concentration of particles from soluble forms or nanoparticles (NPs). More importantly, these technologies could not simultaneously grasp the information of polydisperse NPl including their size, mass and number concentration. In addition, a separation step is imperative before quantitative analysis of NPl to extract polydisperse NPl from biological matrixes, separate them size by size and bring them into a measurable state.

Asymmetric flow field flow fractionation (AF4), as a novel hydrodynamic size-based separation technique, can separate polydiseperse analytes by balancing the diffusion force of the analytes and the external field (cross flow) to keep analytes at unequal velocities (following a parabolic law) within the channel.^27^ As a non-destructive method with considerable separation range and excellent resolution, AF4 could on-line couple with several detectors, *e.g.* a diode array detector (DAD) and multiangle light scattering (MALS). AF4-DAD-MALS has been applied to analyze macromolecules, exosome and NPs.^28–30^ Nevertheless, it is difficult to analyze polydisperse particle in biological matrices due to the complexity of matrix and the heterogeneity of analytes. In this study, a method to separate and detect polydisperse NPl in biological matrix using AF4-DAD-MALS was firstly proposed.

Polystyrene (PS) is one of the five main types of plastic produced, and PS NPs were commonly used as the model of NPl in toxicity studies.^4,31,32^ We selected five sizes of PS NPs (30, 60, 100, 200, 500 nm) successively abbreviated as PS 30 nm, PS 60 nm, PS 100 nm, PS 200 nm, and PS 500 nm as the representative model of NPl. The radius and number concentration were in line with the value supplied by manufacture (Table S1†).

To separate polydisperse PS NPs using AF4, cross flow and detector flow, focus flow and time, and carrier composition and concentration require optimization.^33,34^ The composition and concentration of carrier fluid is undoubtedly crucial and should be carefully investigated firstly. The optimum carrier fluid should avoid the particles agglomeration (obtaining the correct size), particles loss (obtaining high signal) and obtain effective separation among particles (resolution > 1.0).^27^ In this study, five PS NPs could be eluted from small size to large size with time within 25 min by using H_2_O as a carrier fluid (Fig. 1A). However, the void peak and PS 30 nm peak merged together (Fig. S1†), while PS 60 nm, 100 nm, 200 nm and 500 nm were not completely separated (Fig. 1A). For 0.1% (v/v) FL-70 as a carrier fluid, the effective separation of five sizes of PS NPs was achieved (Fig. 1B). However, the UV signal exhibited a high background that would affect the accurate quantification of PS NPs (Fig. S2†). Luckily, the effective separation of five sizes of PS NPs was also achieved by using 0.005% (m/v) SDS, 0.1 mM NaCl, 0.5 mM (NH_4_)_2_CO_3_, or 0.5 mM phosphate buffer (PB) as a carrier fluid (Fig. 1C–F) after optimizing their respective concentrations (Fig. S3†). The radii and UV peak areas of PS NPs using the above four carrier fluids were similar (Fig. 1G and H). Moreover, smooth baselines with low noise background, symmetrical and sharp peaks were acquired by using 0.1 mM NaCl as the carrier fluid. Therefore, 0.1 mM NaCl was selected for further study. Other AF4 parameters *e.g.* cross flow were systematically investigated and discussed (Fig. S4 and S5†). Consequently, the optimum condition was that an initial cross flow of 1.0 mL min^−1^ linearly declined to 0.1 mL min^−1^ in 40 min, detector flow was 0.5 mL min^−1^, and the focus flow and time were 1.0 mL min^−1^ and 5 min, respectively. The optimum eluted program of AF4 was listed in Table S2.† In the optimum condition, the radii ranged 14.0–20.0 nm, 26.0–30.0 nm, 48.0–56.0 nm, 95.0–103 nm, and 232–240 nm, and the average radii were 19.4, 29.4, 52.6, 102, 240 nm for PS 30 nm, PS 60 nm, 100 nm, 200 nm and 500 nm, respectively. The sizes and size distributions of PS NPs after AF4 separation were consistent with their respective monodisperse values (Fig. 2). Furthermore, spherical and uniform particles could be observed after AF4 separation (Fig. S6†), indicating that AF4 is a mild and excellent method for separating polydisperse sample.

**Fig. 1:**
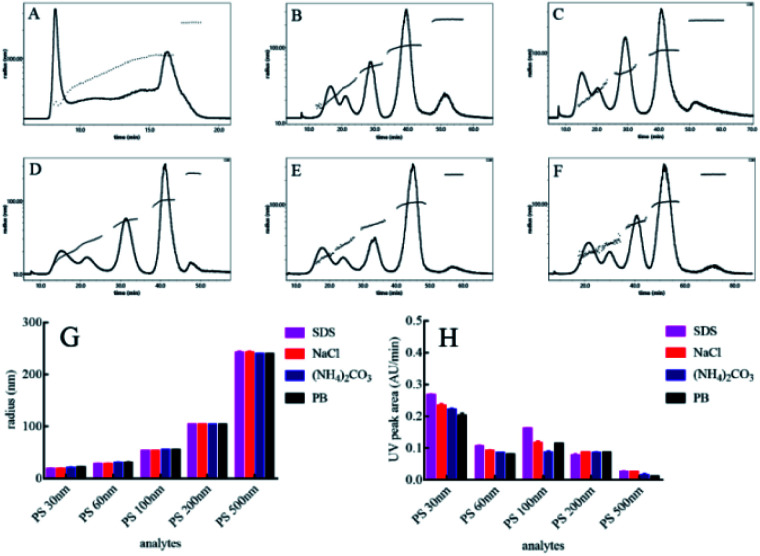
The AF4-MALS fractogram using various carrier fluids (A: H_2_O, B: 0.1% (v/v)FL-70, C: 0.005% (m/v) SDS, D: 0.1 mM NaCl, E: 0.5 mM (NH_4_)_2_CO_3_, F: 0.5 mM PB), radii (G) and UV peak areas (H) of PS NPs.

**Fig. 2:**
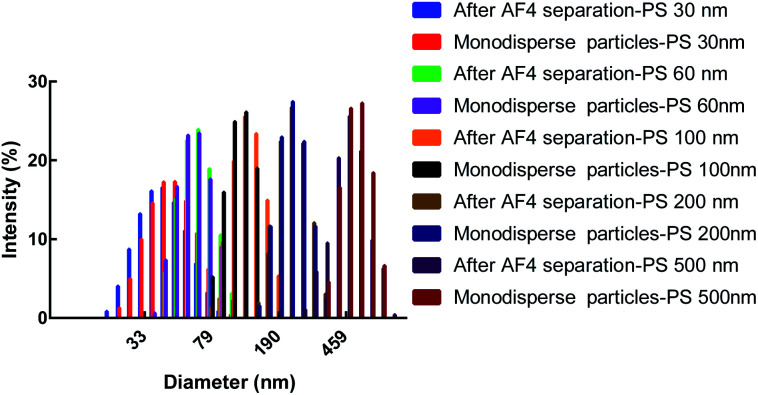
The characterization of five PS NPs after AF4 separation and their respective monodisperse particles by DLS.

After effective separation, five peaks were separated into five fraction (F1–F5) corresponding to PS 30 nm, 60 nm, 100 nm, 200 nm, 500 nm on the basis of the measured radius, and the particles number of different size in each fraction showed in Fig. 3. The particles number of PS 30 nm and 60 nm reduced with the increase of size, and 90% and 84% particles were at 14–18 nm, 26–28 nm. The 87% PS 100 nm, 85% PS 200 nm, and 87% PS 500 nm were in the range of 50–56 nm, 101–103 nm, and 238–240 nm, respectively. The total number of each sized PS NPs and the theoretical number supplied by manufacture were on the same order of magnitude (Table 1). In addition, we evaluated the accuracy of polydisperse particle number quantification using AF4-MALS by injecting different amounts of the mixed PS NPs. The result gave out linear increase in the detected particle number with increasing injection amounts (*R*^2^ > 0.985, Fig. S7†), suggesting that the number of polydisperse PS NPs was closely related with the mass. Therefore, the size, size distribution, and number concentration of polydisperse PS NPs could be accurately measured after effective separation using AF4-MALS.

**Fig. 3:**
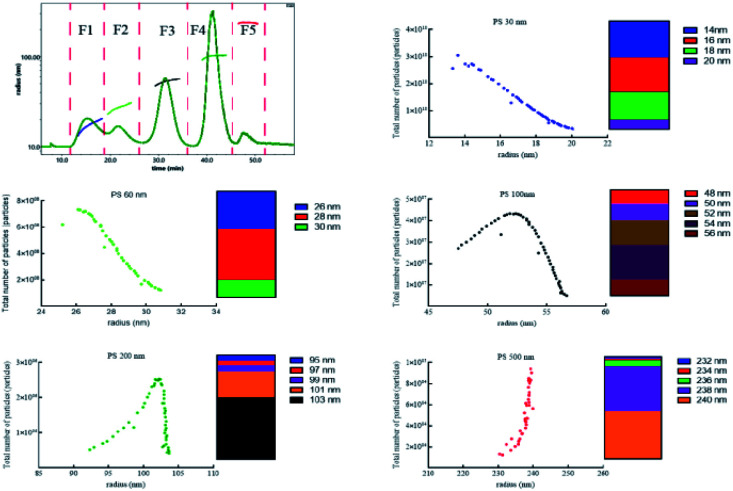
The total number particles *versus* the radius of polydisperse PS NPs in aqueous solution.

**Table 1:** The total number particles of polydispersed PS NPs in aqueous solution after AF4-MALS

Analytes	Total number of particles_theory_	Total number of particles_measure_	SD (particles)
PS 30 nm	9.60 × 10^11^	9.39 × 10^11^	1.48 × 10^10^
PS 60 nm	1.48 × 10^10^	1.98 × 10^10^	3.54 × 10^9^
PS 100 nm	1.52 × 10^9^	1.39 × 10^9^	9.19 × 10^7^
PS 200 nm	8.00 × 10^7^	7.30 × 10^7^	4.95 × 10^6^
PS 500 nm	6.70 × 10^6^	5.7 × 10^6^	7.05 × 10^5^

To separate tiny particles from biological matrix is a very difficult work. Owing to the high surface free energy of NPs, biomolecules bind to the surface of NPs to form a biological coating, known as the protein corona within 0.5 min.^35^ The thickness and density of this protein coating were strongly dependent on the particle size.^36,37^ To achieve the analysis of polydisperse PS NPs in the biological matrix, we further investigated the pre-treatment method compatible with the above proposed AF4-DAD-MALS method. The signals of small particles *e.g.* PS 30 nm and PS 60 nm obtained were low even though using high centrifugal force (20 000 g, Fig. S8A†). In contrast, five PS NPs could be successfully separated from biological matrix using AF4 after alkali digestion (Fig. S8B†). But the radius was higher than its original values, and this difference was gradually reduced with the radius increased (Fig. S9†). We tried to further reduce the biological matrix by increasing the KOH concentration and bathing temperature (Fig. S8C and D†). Finally, the 10% (m/v) KOH and 60 °C were selected to process biological sample. After separation, five peaks could be obviously observed and separated to five fractions (F1–F5). The radii's range of F1–F5 were 24.0–30.0 nm, 35.0–43.0 nm, 52.0–60.0 nm, 95.0–105 nm, and 243–247 nm, and the average radii were 27.4, 35.6, 56.4, 103, 243 nm, corresponding to PS 30 nm, 60 nm, 100 nm, 200 nm and 500 nm, respectively (Fig. 4). The majority of particles ranged 26–28 nm (89%), 35–37 nm (86%), 52–58 nm (87%), 100–105 nm (94%), 242–244 nm (95%) in F1–F5, respectively. In addition to the size, the number of each size particle included in each fraction could be calculated by ASTRA soft according to the signal of MALS. Consequently, the total particles of F1–F5 were 8.61 × 10^10^, 5.48 × 10^9^, 1.89 × 10^9^, 1.19 × 10^8^, 8.15 × 10^6^, respectively. Since the number and radius of each size particle could be obtained simultaneously in biological matrix after effective separation, it would facilitate to accurate study of the toxicity of polydisperse NPl and monitor their exposure level. It was a tiny pity that the radii of PS 30 nm and PS 60 nm obtained was still higher than those detected in aqueous solution. The deviations might be caused by the biological matrix interference. The difference may be minimized by optimizing the pre-treatment method *e.g.* combined use of various digestion solutions. However, another important factor also needed to consider is the low concentration of the particles in the final digestion solution, which makes the applied analytical techniques very difficult.^38^ We will do this work in the future research.

**Fig. 4:**
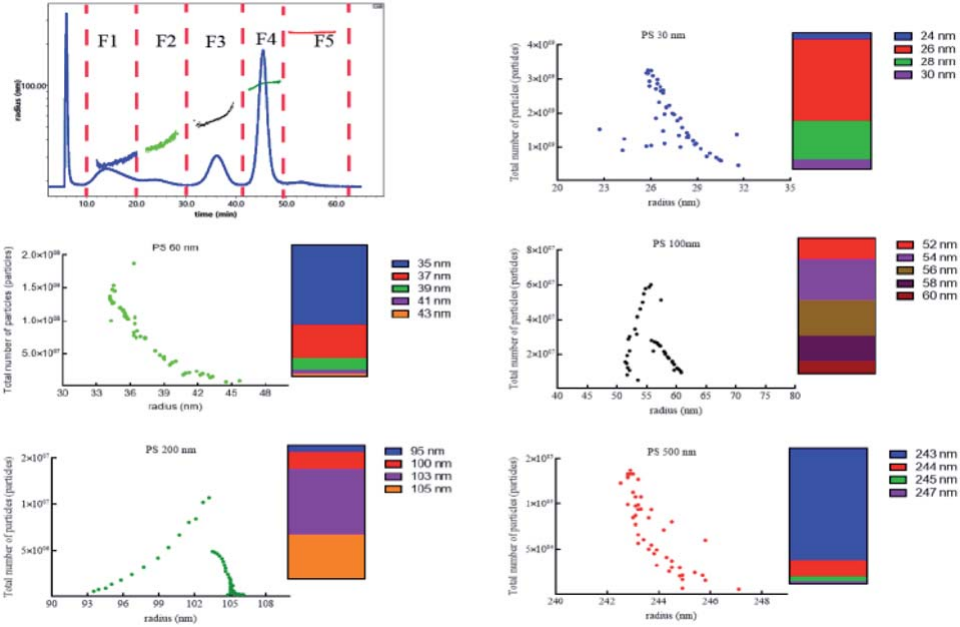
The total number particles *versus* the radius of polydispersed PS NPs on biological solution.

Fortunately, the radius' range, average radius and majority of particles range of PS 100 nm, PS 200 nm and PS 500 nm were consistent with their theoretical values. Moreover, the total number was also close to their respective number for PS 100 nm, 200 nm and 500 nm (Table S3†). Therefore, AF4-MALS could be used to simultaneously separate five PS NPs from biological matrix, and the radius and number of larger particle (≥100 nm) could be accurately calculated.

Generally, the mass concentration of NPl is detected by the mass detector *e.g.* DAD or MS.^39^ Excellent linearity (*R*^2^ > 0.993) was obtained by plotting the UV peak areas (*Y*) *versus* the mass of PS NPs (*X*) in this study (Fig. 5). The intra- and inter-experimental RSDs were 0.32–13.6% and spiked recoveries were 95.6–105%, suggesting the good reproducibility and reliability of the proposed method in quantifying polydisperse PS NPs even in the biological matrix. However, the slope of standard curves were various, PS 200 nm was highest meaning that PS 200 nm was more sensitive than others. This might be related to the special properties of NPs, namely, the detected signal is closely related to its mass concentration and size. Similar phenomenon also had been reported.^20,40,41^ For example, scholars used ICP-MS to quantitatively analyze Au NPs (5 nm, 20 nm and 50 nm), and found that the concentration and signal of Au NPs showed good linearity for each size particle (*R*^2^ 0.99).^42^ However, the 5 nm Au NPs was more sensitive than the other two large particle sizes. Others also found that PS 200 nm had a higher absorbance and much better detectability in comparison with PS 50 nm and PS 100 nm using UV as a detector.^20^ Therefore, in addition the need of effective separation for polydisperse particle, an accurate calibration curve of each size particles was indispensable before quantitative analysis.

**Fig. 5:**
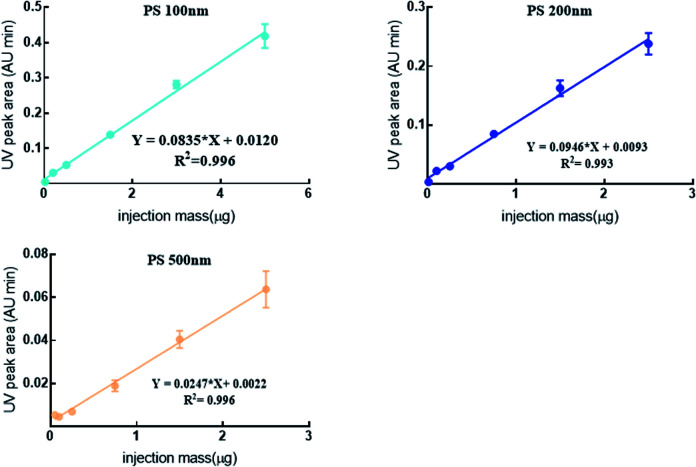
The DAD online quantification standard curve of PS NPs mixture in biological fluid by AF4-DAD-MALS.

However, to draw an accurate calibration curve may become extremely difficult for measuring the mass concentration of unknown particle. Therefore, we further proposed a mass calculation method to estimate the mass concentration of PS NPs as a function of their size and quantity of particle. The volume of particles could be obtained according to the radius, then the mass of particle could be calculated on the basis of *m* = *ρ* × *v* × number of particles (*m* is the calculated mass of particle, *ρ* is the particle density, 1.055 g cm^−3^ for PS NPs, *v* is the volume of particles). The radius and number of every sized particle could be measured by AF4-MALS. The density of particle (*ρ*) is one of the characteristic of particles which could be acquired by identifying the chemical composition of particles through Raman or Fourier transform infrared spectroscopy. Consequently, the RSD between calculated mass and theoretical mass supplied by manufacture was less 11% (Table 2), suggesting that the calculated method based on the radius and number of particles was a good alternative for estimating mass of unknown particles and solving the problem of the absent standard.

**Table 2:** The calculated and theoretical mass of PS NPs

Analytes	Sample matrix					
	Aqueous solution			Biological solution		
	*m*_theory_ (μg)	*m*_calculation_ (μg)	RSD (%)	*m*_theory_ (μg)	*m*_calculation_ (μg)	RSD (%)
PS 30 nm	12.0	12.7	4.01	—	—	—
PS 60 nm	1.60	1.56	1.79	—	—	—
PS 100 nm	0.80	0.93	10.6	1.07	0.92	10.7
PS 200 nm	0.40	0.41	1.75	0.57	0.56	1.25
PS 500 nm	0.40	0.42	3.45	0.57	0.52	6.49

Lastly, the AF4-DAD-MALS method was applied to analyze polydisperse PS NPs in the blood circulation system of rats. As a result, the concentration of PS 100 nm, 200 nm, 500 nm were 1.13 × 10^11^, 2.22 × 10^10^, and 2.59 × 10^9^ particles per mL corresponding to 269 μg mL^−1^, 244 μg mL^−1^ and 65.7 μg mL^−1^ in the whole blood sample collected after exposure to PS NPs 5 min. In comparison with 5 min, the concentration of the three PS NPs declined 93.2%, 88.8% and 50.5% after exposure to 2 h, indicating that PS NPs declined quickly once they entered into blood system. However, whether they were cleared or accumulated in the tissue needed further investigation. Good correlations (*r* > 0.955) were observed between the calculated mass obtained by AF4-MALS and measured mass obtained by AF4-DAD (Fig. 6). Besides, mass of particles from the two methods were further analyzed by the paired-sample Wilcoxon *t*-test, and no significant difference was found (*P* > 0.05). The represented AF4-MALS fractogram of PS NPs was shown in Fig. S10A.† The radius of three PS NPs kept in constant in 2 h (Fig. S10B†), the mass and the total number of three PS NPs declined with the increase of circulated time (Fig. S10C and D†), and spherical with uniform distributions could be observed for three PS NPs by TEM (Fig. S10E†), indicating that PS NPs kept its morphology during penetrate into circulation system.

**Fig. 6:**
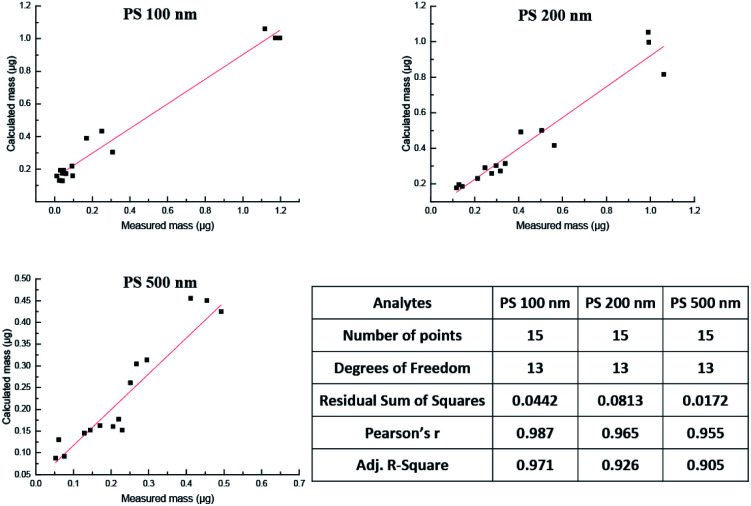
The correlation analysis of PS NPs mass in bio-samples obtained by the calculated method and measured method.

In conclusion, the capability to separate and detect polydisperse PS NPs ranging from 30–500 nm in biological sample has been demonstrated. Polydisperse PS NPs was successfully separated by AF4, and the on-line detection was achieved by coupling AF4 with DAD-MALS. To measure unknown particles in a sample, the calculated mass as function of radius and total number of particles exhibited great potential due to its accuracy and simplicity. Furthermore, the proposed method was applied to separate and detect polydisperse PS NPs in the blood circulation system of rats. It is a pity that the radius and number concentration of small particles (<100 nm) could not be accurately measured. However, this work introduces an effective solution to separate polydisperse PS NPs and detect their size, mass and number concentration of unlabeled particles in biological matrices which will be useful for the accurate study of the pollution level and toxicity of polydisperse NPl. Therefore, taking the excellent separation, simple operation, accurate mass detection and unbiased particle counting into consideration, AF4-DAD-MALS is a promising method for the analysis of polydisperse NPl in biological samples.

## Conflicts of interest

There are no conflicts to declare.

## Supplementary Material

RA-011-D1RA00450F-s001
