# Supplementary material for: An effective solution to simultaneously analyze size, mass and number concentration of polydisperse nanoplastics in a biological matrix: asymmetrical flow field fractionation coupled with a diode array detector and multiangle light scattering
Source: RSC Adv. 2021 Apr 6;11(21):12902–6. doi: 10.1039/d1ra00450f (PMC8697335; doi:10.1039/d1ra00450f)
Supplement: RA-011-D1RA00450F-s001 [file RA-011-D1RA00450F-s001.pdf]

Electronic Supplementary Material (ESI) for RSC Advances.
This journal is © The Royal Society of Chemistry 2021

Electronic Supplementary Information

An effective solution to simultaneously analyze size, mass and number concentration of polydisperse nanoplastics in biological matrix: asymmetrical flow field fractionation coupled with diode array detector and multiangle light scattering

Xing-ling Luo[a], Ying-ting Wu[b], Ling-Yan Zhang[a], Ke-xin Li[a],Tian-jiang Jia[a], Yi Chen[c], Li-hong zhou[a], Pei-li Huang[a]*

a School of Public Health, Capital Medical University, Beijing 100069, China.

b Core facility Center, Capital Medical University, Beijing 100069, China.

c School of Basic Medical Sciences, Capital Medical University, Beijing 100069, China.

*Corresponding Author：

Peili Huang: huangpl@ccmu.edu.cn; Tel.: +86 10 83916539; Fax: +86 10 83911507

Table of Contents

Experimental Section 3

1. Materials and reagents 3

2. Characterization of PS NPs by Dynamic light scatterer (DLS) and Transmission electron microscope (TEM). 3

3. Asymmetrical flow field fractionation-diode array detector-multiangle light scattering detector (AF4-DAD-MALS). 3

4. Optimization of sample pretreatment for biological samples. 4

5. Analysis of polydisperse PS NPs by AF4-DAD-MALS in the whole blood system of rats. 4

Figures 5

Figure S1. The fractogram of five monodisperse PS NPs using ultrapure water as a carrier fluid 5

Figure S2. The blank fractogram (A) and UV signal (B) using different carrier fluids 6

Figure S3. The fractogram of polydispersed PS NPs using different concentration of carrier fluids 8

Figure S4. The fractogram of five PS NPs at different AF4 parameters 10

Figure S5. The orginal and normalized UV peak areas of five PS NPs (*n*=3) 11

Figure S6. Five PS NPs after AF4 separation and the mixture solution composed by five PS NPs detected by TEM. 12

Figure S7. The linearity of the total number versus the mass of PS NPs 13

Figure S8. The fractogram of five PS NPs after using various pretreatment methods. 15

Figure S9. Relative frequency of PS NPs radius obtained on aqueous solution (black) and serum solution (red) 15

Figure S10. The application of AF4-DAD-MALS in the whole blood sample 16

Tables 17

Table S1. The characterization of PS NPs by MALS 17

Table S2. The eluted program of AF4 for five PS NPs 18

Table S3. The mass and total number particles of PS NPs in biological fluid 19

Experimental Section

1. Materials and reagents

Five sizes of polystyrene NPs (30, 60, 100, 200, 500 nm) successively abbreviated as PS 30 nm, PS 60 nm, PS 100 nm, PS 200 nm, and PS 500 nm at a mass concentration of 40 μg/μL were purchased from Thermo Fischer Scientific (Waltham, MA, USA), and stored in the refrigerator at 4 °C. Ultrapure water (resistivity 18.2 MΩ/cm) was collected using a Millipore water purification system (Billerica, MA) and used in all experiments. Chromatographic grade methanol and FL-70 were acquired from Fisher (Ohio, USA). Bovine serum albumin (BSA), human serum albumin (HSA) and sodium dodecyl sulfate (SDS) were purchased from Sigma-Aldrich (St. Louis, MO, USA). Millipore precut regenerated cellulose (RC) membranes with a molecular weight cut-off of 10 kDa and red casein were supplied by Wyatt Technology (Wyatt Technology, USA). Normal rat serum was purchased from Applygen Technologies Inc. (Beijing, China). Sodium chloride (NaCl) was obtained from Beijing Chemical Plant (Beijing, China).

2. Characterization of PS NPs by Dynamic light scatterer (DLS) and Transmission electron microscope (TEM).

The size of PS NPs was determined using a Zetasizer Nano ZS90 (Malvern Instruments Ltd., Germany) at 25 °C in triplicate. Specially, 1 μL monodisperse PS NPs standard solution or PS NPs mixture standard composed by five PS NPs was diluted 1000 times and directly detected by DLS. For TEM analysis, 10 μL of sample solutions were drop-cast on a formvar-coated copper grid and dried in a 37 °C thermotank overnight. TEM of the PS NPs was performed with a JEM-2100 (JEOL, Japan). The fractions (F1-F5) after AF4 separation were collected and detected using DLS and TEM mentioned above.

3. Asymmetrical flow field fractionation-diode array detector-multiangle light scattering detector (AF4-DAD-MALS).

AF4 measurements were performed using an Eclipse Dual tech system (Wyatt Technology, USA) coupled to a 1260 HPLC system (Agilent Technologies, Santa Clara, USA), which contains a quaternary pump, an online vacuum degasser, an autosampler and a DAD. To further ensure a particle-free carrier liquid entered into the AF4 channel, a 0.1 μm membrane filter was placed between the quaternary pump and AF4 channel. Separation of five PS NPs occurred in a trapezoidal-shaped channel which equipped with a 350 μm spacer and an RC 10 kDa membrane as the accumulation wall. The concentrations of the five PS NPs were detected using an online DAD detector operating at 230 nm, while the radii and number of particles were monitored and calculated by using a Dawn Heleos II MALS detector operating at a wavelength of 658 nm. The calibration of the instrument was

performed with a 90° MALS detector using toluene, whereas the other detectors were normalized with BSA as an isotropic scattered standard.

To build good separation conditions for the five PS NPs, several carrier fluids, e.g., ultrapure water, 0.1 % (*v/v*) FL-70, SDS ranged from 0.001 - 0.05 % (*m/v*), NaCl, $(NH_4)_2CO_3$, and PB in the range of 0.05 - 3.00 mM, were studied. Additionally, various initial velocities of the cross flow (at 0.5, 1.0, 2.0 and 3.0 mL/min), elution modes (constant, linear, exponential), and slopes (namely, how quickly the cross flow dropped to 0.1 mL/min; tested conditions: a decrease in the flow rate from 1.0 to 0.1 mL/min within 20, 30, 40 and 50 min), detector (light scattering, LS) flows (0.3, 0.5, 0.8, 1.0 mL/min), focusing flow rates (0.5, 1.0, 2.0 mL/min) and focusing times (5, 10, 20, 40 min), and pH values of solution in the range of 2-11 were carefully investigated. As a result, the carrier fluid (0.1 mM NaCl) was delivered into the AF4 channel by an HPLC pump and equilibrated for 1 h before sample injection. After that, a 20 μL mixed solution of the five PS NPs or sample was injected into the AF4 channel and separated using the optimum eluted program listed in Table S1 at a fixing LS flow rate of 0.5 mL/min.

4. Optimization of sample pretreatment for biological samples.

Several pretreatment methods, including digestion methods using concentrated nitric acid (HNO_3), potassium hydroxide (KOH), and hydrogen peroxide (H_2O_2) as digestion reagents, centrifugation, and ultrafiltration were selected to process biological sample. The influencing factors, e.g., the concentration (10 %, 30 %, 50 % (*w/v*), saturation) of KOH and bathing temperature (60, 80, 100 °C) were investigated.

5. Analysis of polydisperse PS NPs by AF4-DAD-MALS in the whole blood system of rats.

All animal experiments were performed in compliance with the institutional ethics committee regulations and guidelines on animal welfare with the approval of the Animal Experiments and Experimental Animal Welfare Committee of Capital Medical University (ethical review number: AEEI-2016-076). Male Wistar rats that weighed 200−220 g were obtained from Beijing Vital River Laboratory Animal Technology Co., Ltd. (Beijing, China). The rats were housed under normal laboratory conditions with free access to standard rodent food and water. The rats were kept on a 12 h light/dark cycle. After acclimation, 3 male rats were selected as the experimental groups, and 3 male rats were treated as the control group. The experimental group was injected PS NPs mixture solution including 16 μg/μL PS 100 nm and 200 nm, and 8 μg/μL PS 500 nm through a tail vein. The control group was injected with an equal volume of normal saline in the same way. After the dosage, 0.2 mL blood samples were collected in a 0.6 mL centrifuge tube from the tail vein of the rat at three time points (5 min, 30 min, 1h, 2 h, 4h). The concentrations of PS NPs in the whole blood system were determined by AF4-DAD-MALS. The results were further analyzed by SPSS 19.0 software.

Figures

1. The fractogram of five monodisperse PS NPs using ultrapure water as a carrier fluid

The void peak and PS 30 nm peak merged together, while PS 60 nm, 100 nm, 200 nm and 500 nm were not completely separated using ultrapure water as a carrier fluid, indicating that five PS NPs partially overlapped. Therefore, it was not suitable to use ultrapure water as a carrier fluid for separating polydisperse PS NPs.

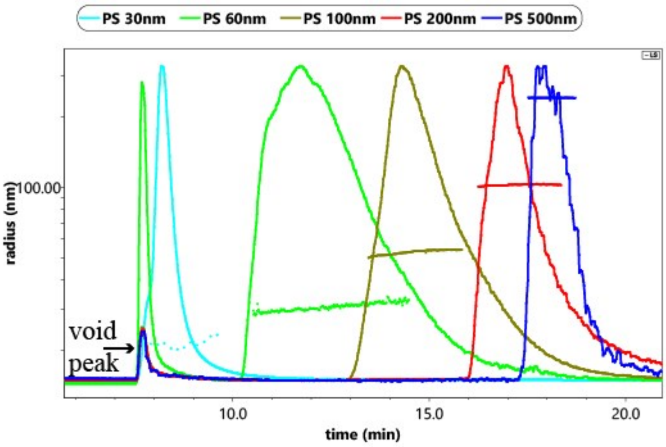

Figure S1. The fractogram of five monodisperse PS NPs using ultrapure water as a carrier fluid

2. The blank fractogram using different carrier fluids.

The MALS signal of blank sample showed highest noisy using SDS as a carrier fluid (Figure S2A), while the UV signal exhibited a high background by using FL-70 as a carrier fluid (Figure S2B). However, both MALS and UV signal showed low background noise using NaCl as a carrier fluid.

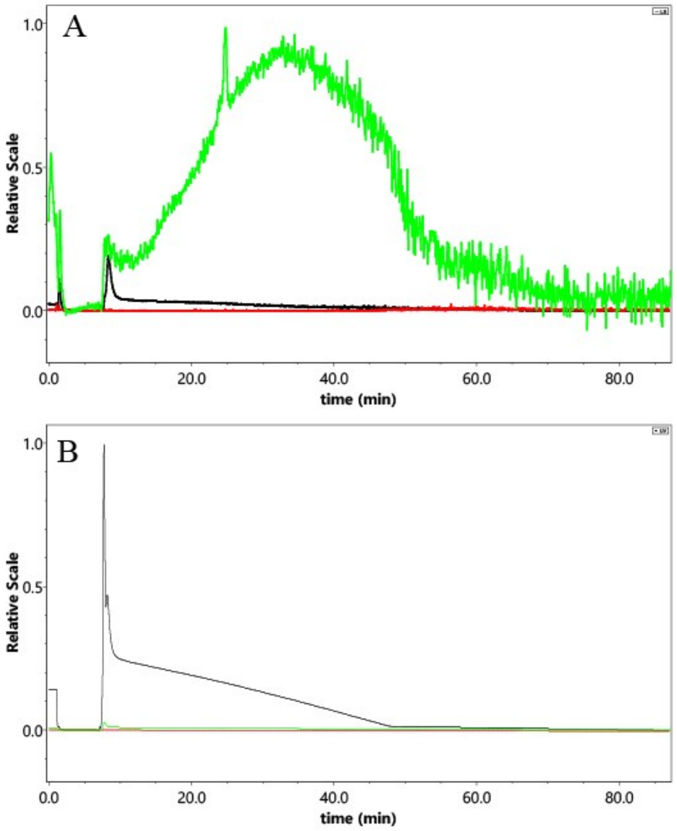

Figure S2. The blank fractogram (A) and UV signal (B) using different carrier fluids

(Black line: FL-70; Red line: NaCl; Green line: SDS)

3. Optimization the optimum concentration of various carrier fluids for separating five PS NPs.

With the concentration of carrier fluids increased, the resolutions among five PS NPs were improved (Figure S3). However, a baseline drift was observed since the concentration of SDS was greater than 0.03 % (*m/v*); and the signals were significantly declined or could not detect at the high concentration of salts used as carrier fluids (> 3.00 mM in this study). Taking the resolution and recovery into comprehensive consideration, the optimum concentration of each carrier fluid was 0.005 % (*m/v*), 0.1 mM, 0.5 mM, 0.5 mM for SDS, NaCl, $(NH_4)_2CO_3$, PB, respectively.

Two hypotheses could be applied to interpret this trend. For one, SDS as an anionic surfactant could form an instant and reversible “simultaneous two-site” adsorption like a bridge between the PS NPs particles and the RC membrane. To be specific, one end of SDS could adsorb on the surface of PS NPs, and the other end interacted with the surface of the membrane. Therefore, PS NPs was instantly adsorbed on the membrane surface, entailing a slowdown in the elution of the nanoparticles. As the concentration of SDS enhanced, the force of the “bridge” increased, so that the interaction between the PS NPs and membrane was gradually improved. Consequently, long elution time of particles with improved resolution and low peak areas were obtained with the added concentration of SDS. For another, NaCl, $(NH_4)_2CO_3$, PB as salted carrier fluids, the compression of electrical double layers could be exploited to expound this phenomenon. Specifically, as the ionic strength increases, the electrical double layer particles were compressed inch by inch, resulting in a mild decrease in the PS NPs radii. With increasing NaCl, $(NH_4)_2CO_3$, PB concentrations, the PS NPs was closer to the membrane surface and located at slower flow rate lines with the increased concentration of carrier fluid. Then, PS NPs would experience stronger attractive forces and be more likely to be absorbed by membrane. These results were consistent with t literature reports[1].

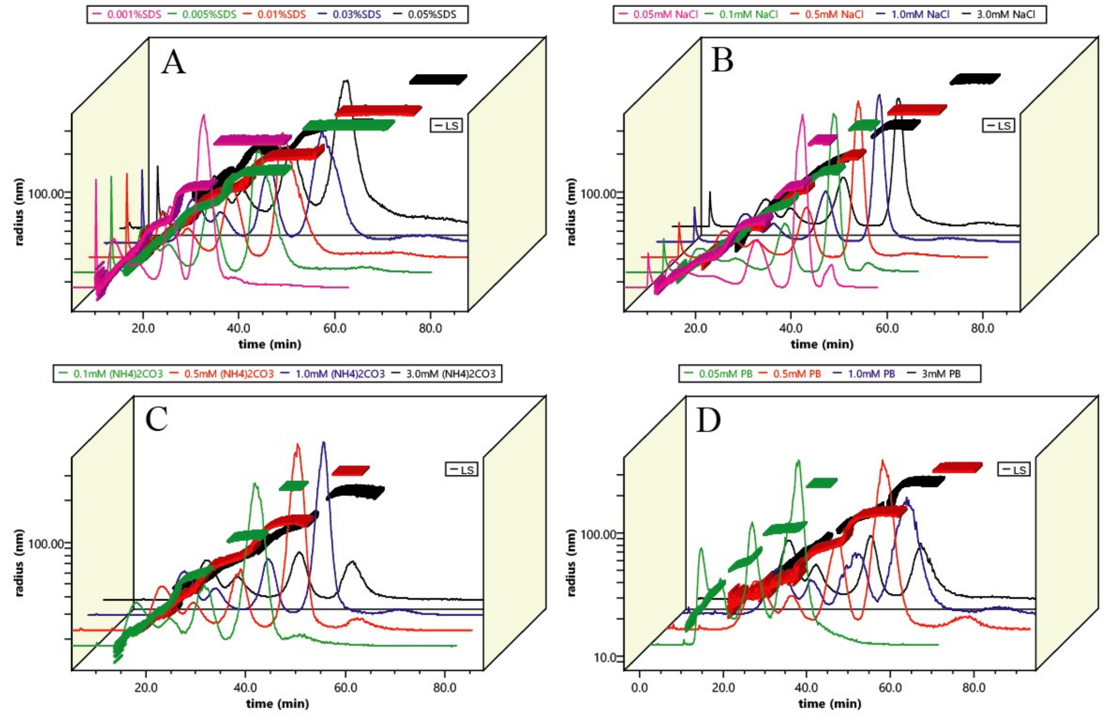

Figure S3. The fractogram of polydispersed PS NPs using different concentration of carrier fluids

(A: SDS, B: NaCl, C: $(NH_4)_2CO_3$, D: PB)

4. The optimization of AF4 parameters for five PS NPs separation.

The cross flow velocity remained constant at 1.0 mL/min (constant elution mode), PS 500 nm could not be eluted within 100 min, and burr peaks could be observed, while the resolution between PS 30 nm and PS 60 nm was less than 1.0 using the exponential elution mode (Figure S4A). Simultaneously, with an increase in the initial velocity of cross flow, the retention time of the five PS NPs was prolonged, and the peak shape became chunky, whereas the PS 30 nm peak was close to the void peak, and the resolution of PS 30 nm and 60 nm was less than 1.0 by applying 0.5 mL/min as the initial cross flow velocity (Figure S4B). Furthermore, the cross flow velocity declined from 1.0 mL/min to 0.1 mL/min in 40 min, and the resolutions among the five PS NPs were larger than 1.0 (Figure S4C). The signals of five PS NPs increased greatly, and the elution time was largely prolonged with a decrease in the LS flow (Figure S4D). However, previous research has predicted that extremely slow elution, e.g., below 0.5 mL/min, would result in low recovery due to the immobilization of a significant quantity of the sample in the channel[2]. In other words, as the LS flow rate decreases, the analyte will be lost on the membrane, resulting in a low detection signal. The paradoxical phenomenon might relate to the dilution effect. Namely, more carrier fluid would be pumped into channel and the sample would be largely diluted using high LS flow velocity e.g. 1.0 mL/min in comparison with low LS flow. If only the dilution effect caused signal difference, the signal obtained in various LS flows would be equal after normalization. To normalize the LS flow, the law of dilution was used according to equation：$C1 \times V1 = C2 \times V2$ (*C1* and *C2* were original and normalized UV peak area, while *V1* and *V2* were the original and normalized LS flow (1.0 mL/min was selected as the normalization LS flow in this study). As a result, the UV peak areas were similar in various LS flows after normalization for PS 30, 60, 100 and 200 nm, but showed a slightly increased trend with the increase of LS flow for PS 500 nm (Figure S5).

For the focus flow and time, no significant changes could be observed, except the void peak was reduced with the focus flow added, and the peak area slightly declined with the focus time increased (Figure S5E and S5F). To investigate the sample solution pH from 2 to 11, the five PS NPs could be separated and distinguished in general (Figure S5G). Consequently, the optimal cross flow parameter was an initial cross flow of 1.0 mL/min and a linear decrease to 0.1 mL/min in 40 min, the LS flow was fixed at 0.5 mL/min, and the focus flow and time were 1.0 mL/min and 5 min, respectively. The sample pH was not required to control.

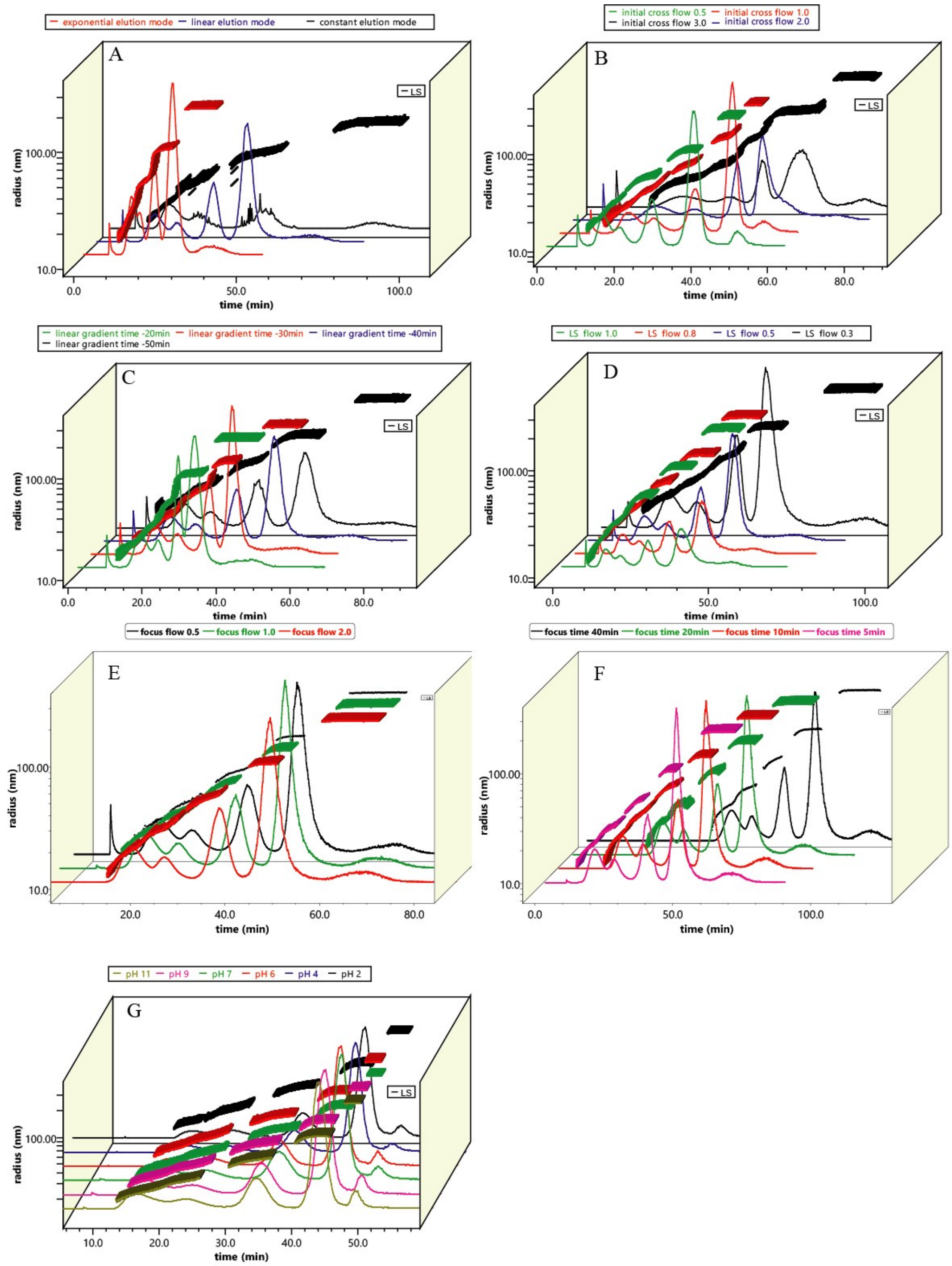

Figure S4. The fractogram of five PS NPs at different AF4 parameters

(A elution modes; B initial velocities of the cross flow; C slopes of the cross flow; D LS flows; E focus flow; F focus times; G pH values of solution)

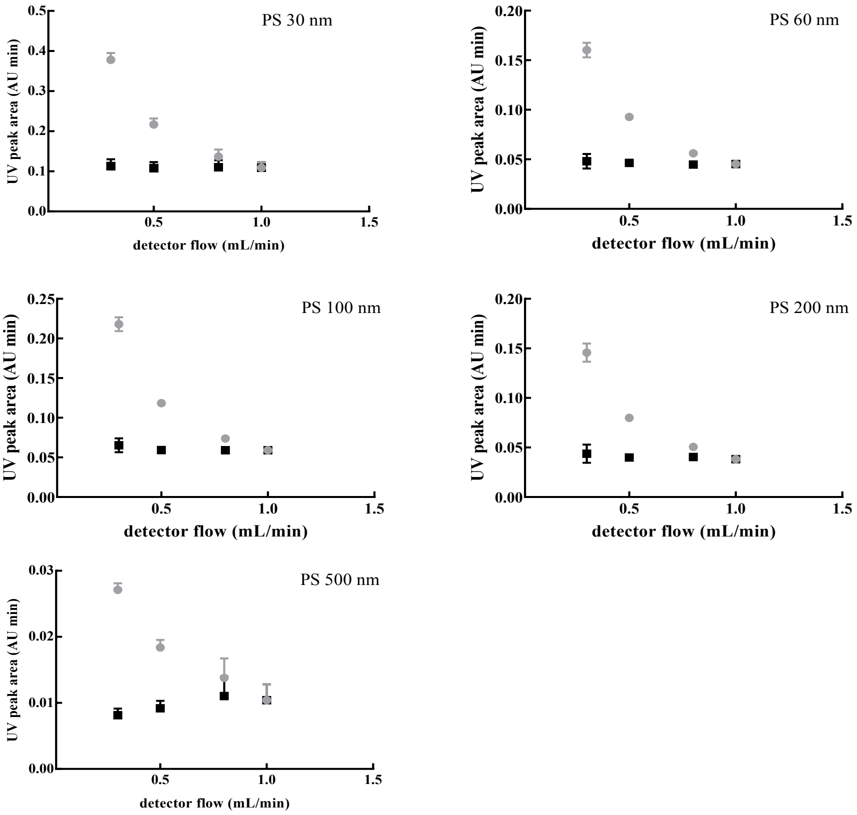

Figure S5. The orginal and normalized UV peak areas of five PS NPs (*n*=3)

(The grey circles represented the original UV peak of PS NPs, while the back squares were the normalized UV peak of PS NPs)

5. The characterization by TEM before and after AF4 separation

After AF4 separation, the mixture solution composed by five PS NPs was separated to five fractions corresponding to PS 30 nm, 60 nm, 100 nm, 200 nm, 500 nm. The spherical and uniform particles involved each fraction could be observed after AF4 separation by TEM. However, differently sized PS NPs could be observed before AF4 separation

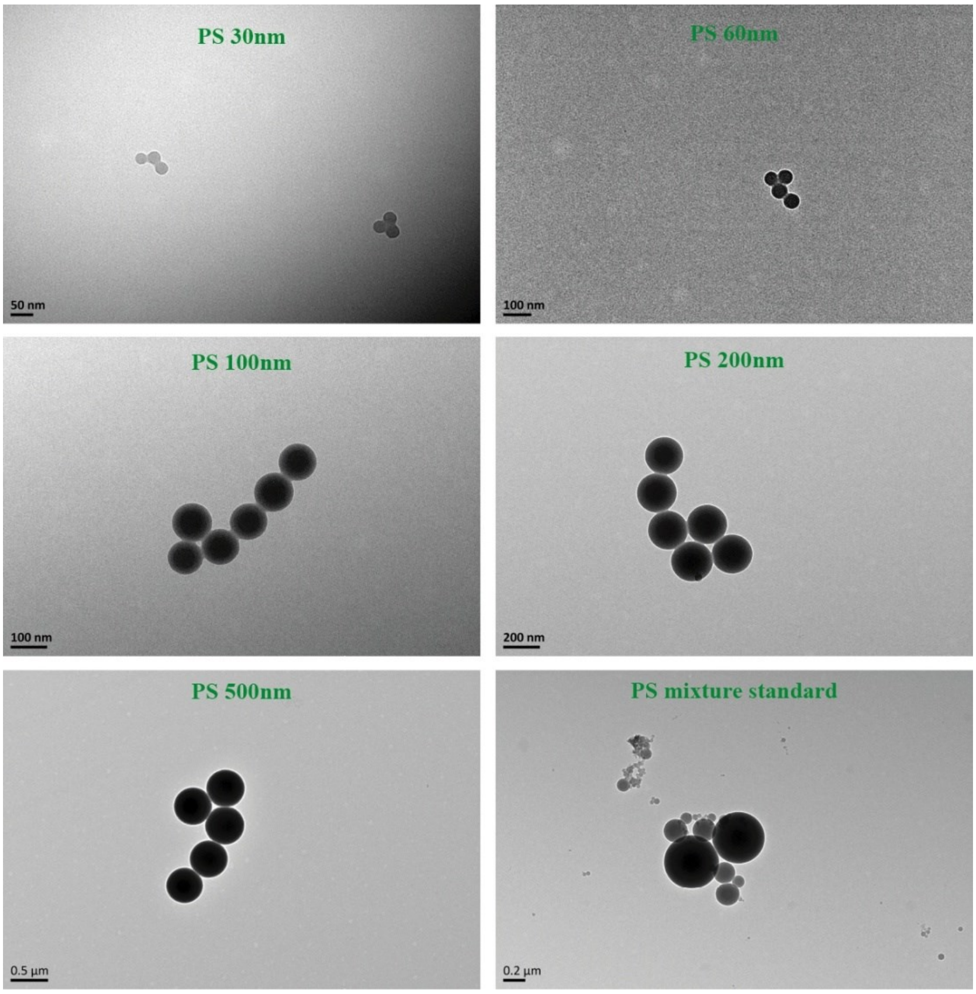

Figure S6. Five PS NPs after AF4 separation and the mixture solution composed by five PS NPs detected by TEM.

6. The relationship between the total number and mass of PS NPs.

The total number versus the mass of PS NPs showed a good linearity (R^2 > 0.985), suggesting that the total number of particles is closely related to the mass of particles in the sample.

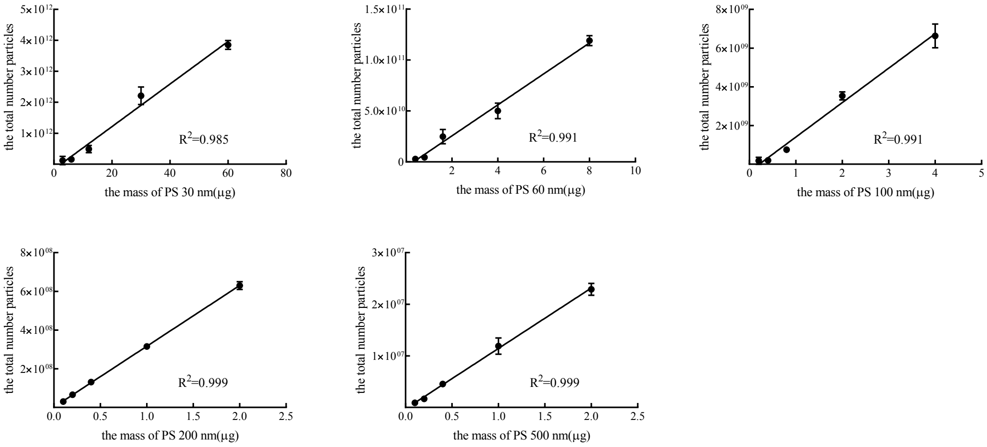

Figure S7. The linearity of the total number versus the mass of PS NPs

7. Optimization of pretreatment for analyzing polydisperse PS NPs in the biosamples.

For analyzing polydisperse NPl in biological samples, the crucial step is to screen a time- and cost-effective pretreatment method that efficiently reduces the impact of the biological matrices without degrading the NPl, as well as without affecting the subsequent use of the AF4-DAD-MALS method. In this study, the five of PS NPs in biological fluid could not be effectively separated and detected by AF4-DAD-MALS after using ultrafiltration, acid or peroxide digestion as pretreatment methods (Figure S8A), whereas the five PS NPs could be clearly observed and separated after centrifugation or alkali digestion (Figure S8B). Notwithstanding, centrifugation could greatly reduce the content of biological macromolecules, the low recovery of small particles such as PS 30 nm and PS 60 nm (even though using high centrifugal force (20000 g)), the requirement of special instruments limited the centrifugation usage. Digestion method can also reduce biological macromolecules. However, HNO_3 possesses strong digestion ability and could directly digest and destroy both biological macromolecules and PS NPs, which reflected that no signal could be detected after acid digestion. Researchers had also reported that the recovery of PS 568 nm was only 1.4 % after HNO_3 digestion[3]. Simultaneously, dense foam was formed and spilled over the container after the addition of hydrogen peroxide, which hampered the filtration and further separation and detection of the samples. A similar phenomenon has been reported[4, 5]. Fortunately, KOH, as a milder chemical digestion reagent, could digest biological macromolecules, which ensures the subsequent separation and detection of PS NPs by AF4-DAD-MALS. Researchers also only recommended these experimental conditions for efficiently digesting biological tissues with no significant degradation of the microplastics[6]. Although five PS NPs could be successfully separated from biological matrix using AF4 after alkali digestion, the radius was higher than its original values, and this difference was gradually reduced with the radii improved, which might be related to protein corona (Figure S9). We tried to further reduce the protein corona by increasing the KOH concentration and bathing temperature. Nevertheless, the signal declined with an increase in the concentration of KOH or bath temperature (Figure S8C and 8D). Therefore, 10 % (*m/v*) KOH and 60 °C were selected to process biological sample.

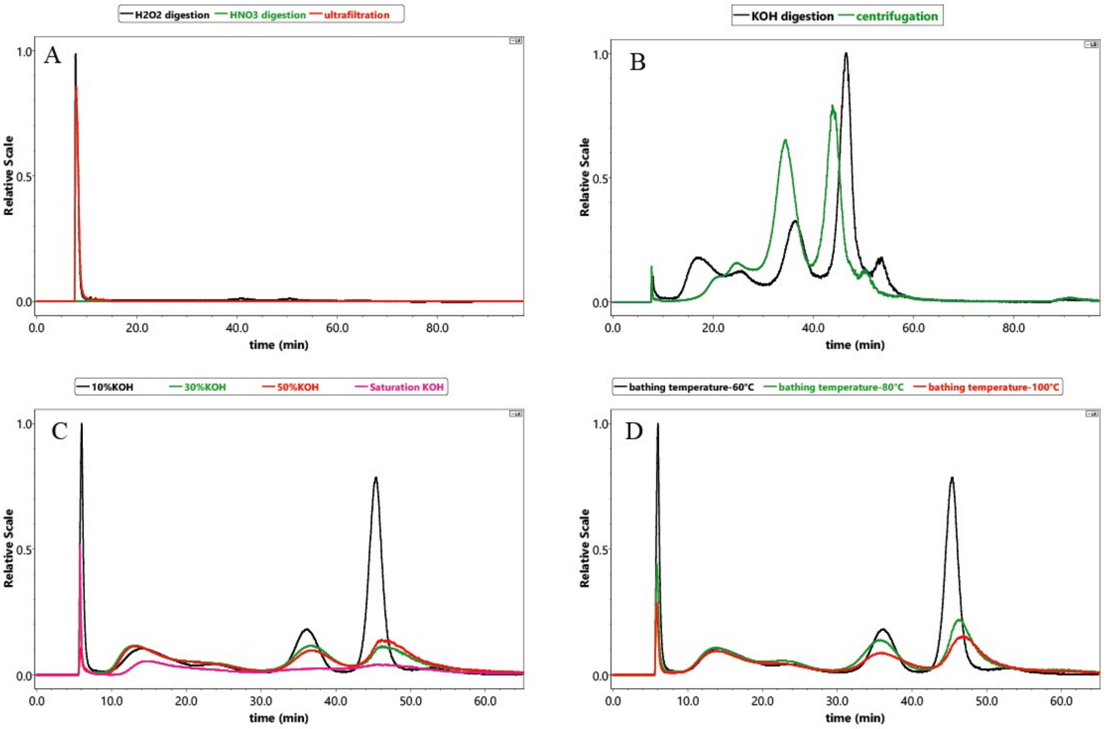

Figure S8. The fractogram of five PS NPs after using various pretreatment methods.

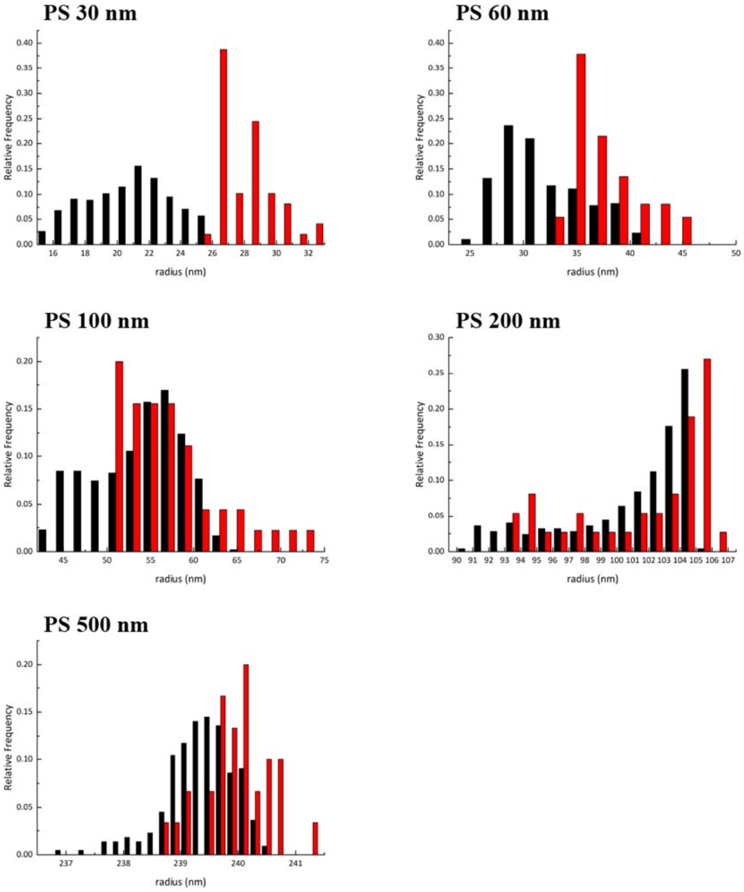

Figure S9. Relative frequency of PS NPs radius obtained on aqueous solution (black) and serum solution (red)

8. The TEM of PS 100 nm (A) and PS 200 nm (B) separated from blood system of rat after exposure to PS NPs.

The AF4-MALS showed the good separation of three PS NPs from the blood system of rat (A). The radius kept constant during the circulation time (B). The mass and total number of PS NPs declined with the time (C and D). The TEM of PS NPs were found to be spherical with uniform distributions, and the radii were close their own actual values (E).

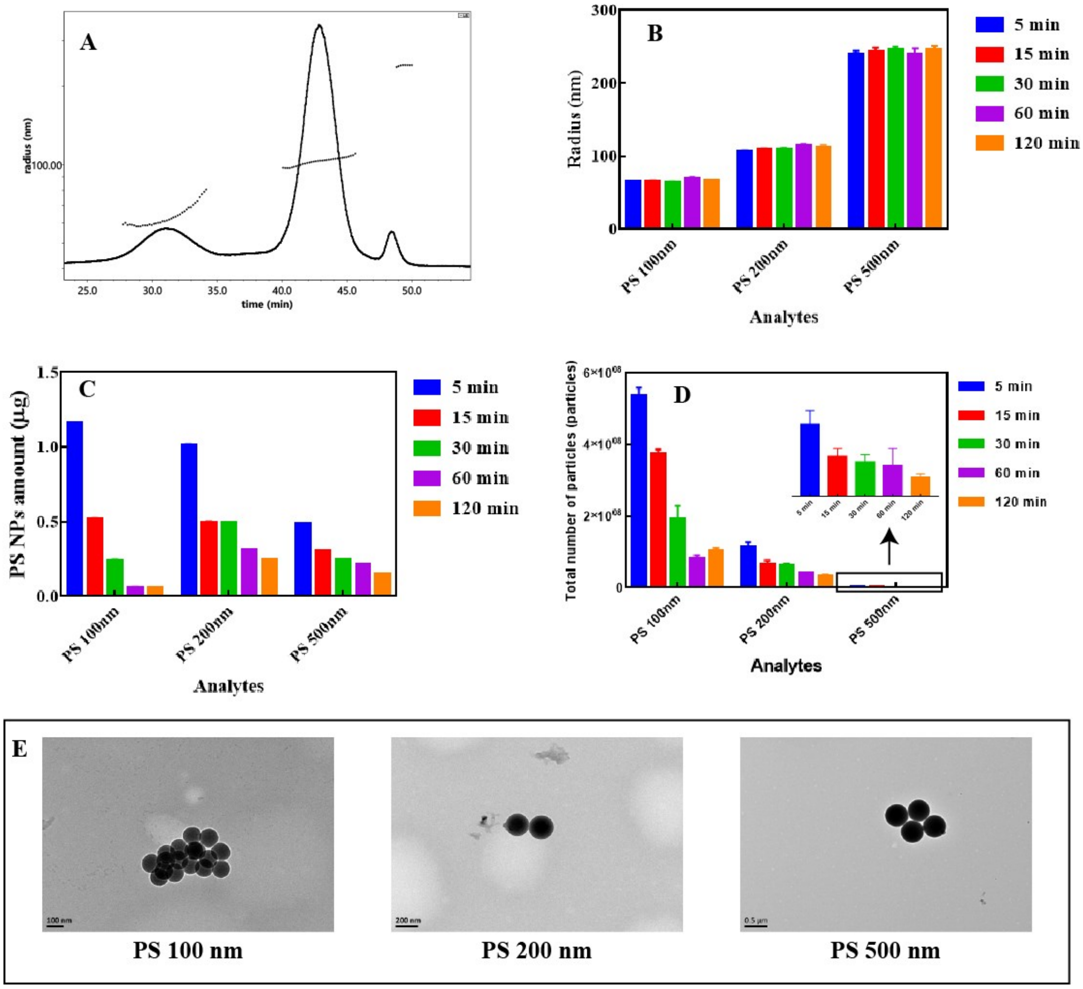

Figure S10. The application of AF4-DAD-MALS in the whole blood sample

(A The AF4-MALS fractogram of PS NPs; B The radius of PS NPs; C The mass of PS NPs;

D The total number particles of PS NPs; E The TEM of PS NPs)

Tables

1. The characterization of PS NPs by MALS

The radii of PS 30 nm, PS 60 nm, PS 100 nm, PS 200 nm and PS 500 nm detected by MALS were 19.4 nm, 29.4 nm, 55.1 nm, 114 nm, 238 nm, respectively. These values were close to the radius supplied by manufacture (SD < 4.3 nm). The total number of PS NPs and their values supplied by manufacture were on the same order of magnitude. Therefore, MALS could be used to accurately detect the size and number of particles.

Table S1. The characterization of PS NPs by MALS

Analytes	r^a (nm)	$r_{measure}$ (nm)	SD (nm)	Total number of particles (particles)[a]	Total number of particles $_{measure}$ (particles)	SD (particles)
PS 30nm	14.0	19.4	3.8	9.60×10^{11}	9.02×10^{11}	4.10×10^{10}
PS 60nm	27.5	29.4	1.3	1.48×10^{10}	1.23×10^{10}	1.77×10^{9}
PS 100nm	49.0	55.1	4.3	1.52×10^{9}	1.18×10^{9}	2.40×10^{8}
PS 200nm	100	104	2.8	8.00×10^{7}	8.30×10^{7}	2.12×10^{6}
PS 500nm	240	238	1.4	6.70×10^{5}	7.58×10^{5}	6.22×10^{4}

[a] supplied by manufacture

2. The optimum eluted program of AF4 for five PS NPs

To be specific, the step 1 and 2 were applied to pre-balance system, step 3 to 5 for separation and elution of target analytes, while the last 5 steps were used to wash the channel and injection loop without cross flow. After running this program, the next sample could be injected immediately.

Table S2. The eluted program of AF4 for five PS NPs

Step	Mode	Duration (min)	Cross flow start (mL/min)	Cross flow end (mL/min)	Flow profile
1	elution	1	1.0	1.0	constant
2	focus	1	1.0	1.0	constant
3	focus injection	5	1.0	1.0	constant
4	elution	40	1.0	0.1	linear
5	elution	- [a]	0.1	0.1	constant
6	elution injection	5	0	0	constant
7	focus injection	5	1.0	1.0	constant
8	elution injection	10	0	0	constant
9	focus injection	3	1.0	1.0	constant
10	elution injection	20	0	0	constant

[a] The time of this step depended on the experimental condition.

3. The mass and total number particles of PS NPs in biological fluid.

The theoretical numbers of particle and the measured value were on the same order of magnitude for larger particles (> 100 nm), while the significant difference could be observed for small particle (PS 30 nm and 60 nm). This might be related to the radius of PS 30 nm and 60 nm was far from their respective values because a 2 % error in the size presented will result in 6 % error in the number calculated[7]. Consequently, the *RSD* between calculated mass and theoretical mass was 21% and 60 % for PS 30 nm and 60 nm, respectively. However, the RSD was less 11 % for other three particles because their radius and number was close to their respective values, meaning that this method still could be used to quantitatively calculate the mass of large particles even in biological matrix.

Table S3. The mass and total number particles of PS NPs in biological fluid

Analytes	m_{theory} (μg)	$m_{calculation}$ (μg)	*SD* (μg)	Total number of $particles_{theory}$	Total number of $particles_{measure}$	*SD* (particles)
PS 30 nm	16.4	12.4	2.83	1.28×10^{11}	8.61×10^{10}	2.96×10^{10}
PS 60 nm	2.03	0.81	0.86	1.97×10^{10}	5.48×10^{9}	1.01×10^{10}
PS 100 nm	1.07	0.92	0.11	2.03×10^{9}	1.89×10^{9}	9.90×10^{7}
PS 200 nm	0.57	0.56	0.01	1.07×10^{8}	1.19×10^{8}	8.49×10^{6}
PS 500 nm	0.57	0.52	0.04	8.93×10^{6}	8.15×10^{6}	5.52×10^{5}

References

1. S. T. Kim, K. Rah and S. Lee, *Langmuir the Acs Journal of Surfaces & Colloids*, 2012, **28**, 10672-10681.
2. J. Gigault, J. M. Pettibone, C. Schmitt and V. A. Hackley, *Anal Chim Acta*, 2014, **809**, 9-24.
3. X. Jiang, L. Tian, Y. Ma and R. Ji, *Sci Total Environ*, 2019, **655**, 591-597.
4. C. G. Avio, S. Gorbi and F. Regoli, *Mar Environ Res*, 2015, **111**, 18-26.
5. M. Claessens, L. Van Cauwenberghe, M. B. Vandegehuchte and C. R. Janssen, *Mar Pollut Bull*, 2013, **70**, 227-233.
6. A. Dehaut, A.-L. Cassone, L. Frère, L. Hermabessiere, C. Himber, E. Rinnert, G. Rivière, C. Lambert, P. Soudant, A. Huvet, G. Duflos and I. Paul-Pont, *Environmental Pollution*, 2016, **215**, 223-233.
7. W. M. Wyatt PJ, inventors, *US Pat. 6,774,994 B1*, 2004, 1-13.
